# Structural Landscape of the Transition from an ssDNA Dumbbell Plus Its Complementary Hairpin to a dsDNA Microcircle Via a Kissing Loop Intermediate

**DOI:** 10.3390/molecules26103017

**Published:** 2021-05-19

**Authors:** Alberto Mills, Federico Gago

**Affiliations:** Área de Farmacología, Departamento de Ciencias Biomédicas, Unidad Asociada al IQM-CSIC, Universidad de Alcalá, Alcalá de Henares, 28805 Madrid, Spain; alberto.mills@edu.uah.es

**Keywords:** DNA, microcircle, dumbbell, hairpin, kissing loop, molecular dynamics

## Abstract

The experimental construction of a double-stranded DNA microcircle of only 42 base pairs entailed a great deal of ingenuity and hard work. However, figuring out the three-dimensional structures of intermediates and the final product can be particularly baffling. Using a combination of model building and unrestrained molecular dynamics simulations in explicit solvent we have characterized the different DNA structures involved along the process. Our 3D models of the single-stranded DNA molecules provide atomic insight into the recognition event that must take place for the DNA bases in the cohesive tail of the hairpin to pair with their complementary bases in the single-stranded loops of the dumbbell. We propose that a kissing loop involving six base pairs makes up the core of the nascent dsDNA microcircle. We also suggest a feasible pathway for the hybridization of the remaining complementary bases and characterize the final covalently closed dsDNA microcircle as possessing two well-defined U-turns. Additional models of the pre-ligation complex of T4 DNA ligase with the DNA dumbbell and the post-ligation pre-release complex involving the same enzyme and the covalently closed DNA microcircle are shown to be compatible with enzyme recognition and gap ligation.

## 1. Introduction

DNA circularization results from the covalent bonding of the two ends of the same linear DNA molecule. The formation of a phosphodiester bond between juxtaposed 5′ phosphate and 3′ hydroxyl termini in double-stranded DNA (dsDNA) is catalyzed by DNA ligases. An enzyme commonly used in laboratory research is bacteriophage T4 DNA ligase, which does not join single-stranded nucleic acids but efficiently joins blunt and cohesive ends and repairs single-stranded nicks in duplex DNA, RNA or DNA/RNA hybrids [1]. Depending on the DNA molecule undergoing cyclization, single-stranded DNA (ssDNA) and dsDNA circles can be obtained. Widely different sizes of circular DNAs are naturally found in viruses, as well as in prokaryotic and eukaryotic cells, where they carry out multiple functions. In fact, the single chromosome that is typically present in bacteria and the extrachromosomal plasmids that are used to transfer genetic information from one individual to another are circular dsDNA molecules. In higher eukaryotes, circular dsDNA can be found in plant chloroplasts and animal mitochondria [2].

Circular DNAs present in nature exhibit attractive properties that have been amply exploited in biotechnology; two of their most outstanding applications are the use of plasmids for molecular cloning and the development of phage therapies. On another front, circular ssDNAs are routinely employed in the creation of three-dimensional (3D) nanostructures known as DNA origami [3]. Whereas natural circular DNAs are enormous in size, the number of base pairs (bp) in artificially created circular DNAs can be easily tuned. Different techniques have been used to create both circular ssDNAs and dsDNAs by means of either chemical or enzyme-mediated ligation of linear nucleic acids [2]. Amongst circular ssDNAs, dumbbells are one of the most interesting structures because of their exceptional usefulness in, for example, DNA amplification [4] and gene therapy [5]. A DNA dumbbell consists of a self-paired duplex stem with two loops made up of unpaired bases at both ends. These unique DNA structures are characterized by (i) increased melting transition temperatures, (ii) improved resistance to degradation by nucleases [2,6], and more readily cell penetration compared to their linear counterparts in a standard DNA double helix [7].

To covalently close a dsDNA into a circular structure using enzyme-mediated ligation, the DNA blunt or cohesive ends must be properly aligned and close enough for the ligase to seal the nick [8]. Although multiple sizes can be obtained in these cyclization assays, the shorter the length of the DNA, the more difficult it is for circularization to be successful and the more likely the appearance of kinks and other disruptions in the double helix [9,10,11]. Nonetheless, different approaches have been developed in research laboratories to create dsDNA minicircles of <100 bp in length. The elegant Ligase-Assisted Minicircle Accumulation (LAMA) assay has allowed the creation of dsDNA minicircles ranging from 63 bp to 205 bp [10,12]. Due to the strong bending needed to close a dsDNA into a circle, the overall structure of the double helix is likely to be subjected to both bending and torsional stress. In fact, experimental and modeling results have demonstrated the presence of different kinds of disruptions, including writhing and formation of kinks and bubbles [9,13,14,15,16].

**Figure 1:**
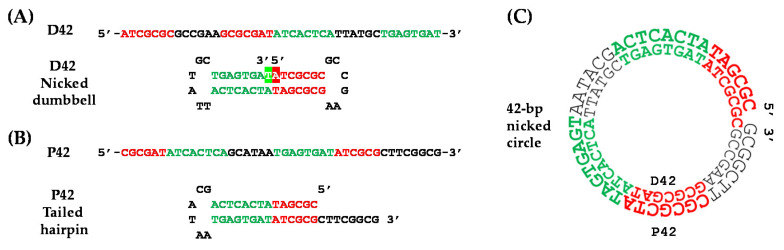
Sequence and schematic representation of the (**A**) D42 and (**B**) P42 ssDNA oligonucleotides that were used in the ligation and circularization experiments to give rise to a (**C**) 42-bp dsDNA microcircle and have been simulated in the present work.

Strikingly, the smallest dsDNA circle described to date is—to the best of our knowledge—the 42-bp microcircle constructed by Wolters and Wittig [17] following a stylish multistage strategy. Two complementary ssDNA oligonucleotides of 42 nucleotides each (D42 and P42) were designed in such a way that the D42 sequence presented an inverted repeat, which led to the formation of a dumbbell structure with a nick in the center of its stem, whereas the P42 oligonucleotide gave rise to a 3′-tailed hairpin (Figure 1). Firstly, the 5′- and 3′-ends of the D42 dumbbell were covalently joined by T4 DNA ligase. Thereafter, this dumbbell was incubated in the presence of P42 to allow hybridization of both complementary sequences and formation of a 42-bp microcircle containing a single nick, which was then sealed by T4 DNA ligase in the last step of the procedure. Treatment with different enzymes throughout the process corroborated the presence of the 42-bp dsDNA microcircles, and a tentative regular DNA model with 10.5 bp per helical turn was proposed [17]. The conformation of such a small circular dsDNA molecule, however, is very unlikely to coincide with that of a canonical B-DNA double helical structure because of the highly bent backbones and the huge bending stress introduced upon formation of the covalently closed microcircle [18]. In fact, it is intriguing—and not easily envisioned—how T4 DNA ligase can recognize and seal both the D42 dumbbell and the 42-bp dsDNA microcircle containing a single nick. Moreover, since little is known about the pre-annealing complex formed between the D42 dumbbell and the P42 hairpin (whose existence must precede that of the dsDNA microcircle), we hypothesized that this process is likely to happen through a kissing-loop complex. The kissing loop is a special type of interaction that takes place between complementary ssDNA loop regions of two dumbbells [19] or, as proposed here, between one of the two ssDNA loop regions of the D42 dumbbell and the ssDNA tail of the P42 hairpin.

Given this background, we thought it would be of general interest to provide insight into the distinct features of all the 3D structures involved in the creation of this 42-bp dsDNA microcircle, namely: (i) the D42 dumbbell, (ii) the P42 hairpin, (iii) the putative pre-annealing complex, (iv) the DNA:ligase complexes, and (v) the final singly nicked and covalently closed 42-bp dsDNA microcircles.

## 2. Results and Discussion

We first modeled in atomic detail and simulated using unrestrained molecular dynamics (uMD) the complementary D42 and P42 ssDNA sequences (42 nucleotides each) that eventually lead to formation of the 42-bp dsDNA microcircle [17] (Figure 1).

### 2.1. D42 Dumbbell and P42 Hairpin

**Figure 2:**
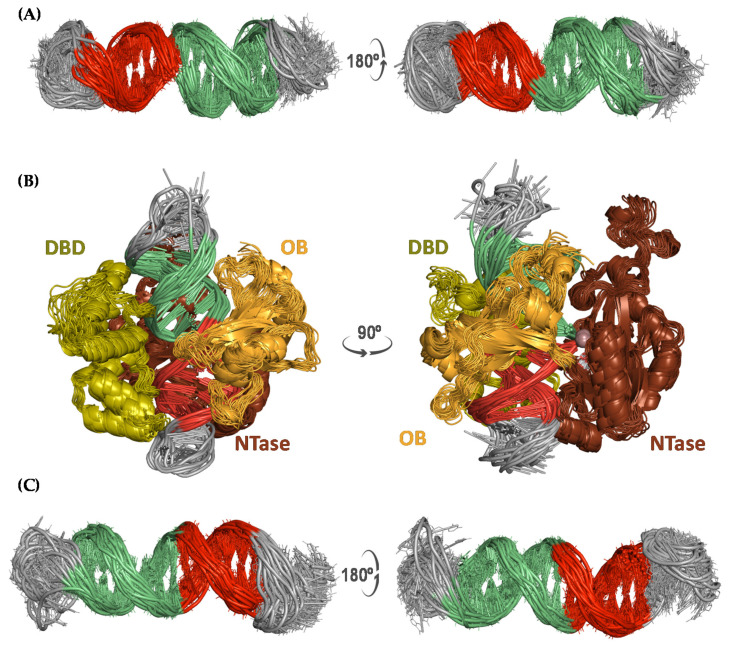
Cartoon representation of an ensemble of 40 structures from two viewpoints (left and right) taken from the uMD trajectories of (**A**) the nicked dumbbell, (**B**) the nicked dumbbell in complex with T4 DNA ligase, and (**C**) the hairpin with a dangling 3′-end. Snapshots separated by 5 ns were obtained from the post-equilibrated 100–300 ns interval of each trajectory. Self-complementary strands are colored in red and green whereas single-stranded regions are colored in grey. The three T4 DNA ligase domains are labeled as DBD (DNA binding domain, residues 1─129), OBD (oligonucleotide-binding domain, residues 370─487) and NTD (nucleotidyl-transferase domain, residues 133─367), and colored in green, orange and brown, respectively. The Mg^2+^ ion is shown as a violet sphere.

The D42 and P42 ssDNA molecules were modeled and simulated for 300 ns (in triplicate) as a nicked dumbbell and a tailed hairpin, respectively, to provide detailed 3D information on these particular DNA structures. The D42 sequence consists of two inverted repeats of seven and eight nucleotides that make it possible for the oligonucleotide—upon pairing of the complementary bases—to bend and form a double-stranded stem containing a single nick; the two loops on both sides of the stem are made up of six unpaired bases that are located between these inverted repeats in the primary sequence (Figure 1A). Simulations of the nicked dumbbell consistently showed the stem structured as a standard double helix and both loops adopting a more disordered, albeit stable, architecture (Figure 2A), as assessed by both visual inspection and low root-mean-square deviations (rsmd) of these regions during the simulation time (Figure S1). Remarkably, the 5′- and 3′-ends of the nick remained suitably aligned during the whole trajectory, which is an essential requirement for the ligase to be able to seal the phosphodiester backbone. The fact that the nick is placed in the middle of the double helical stem is crucial for recognition of this ssDNA by T4 DNA ligase, as it resembles a canonical dsDNA helix. The pre-ligation complex formed between the nicked D42 dumbbell and T4 DNA ligase showed a remarkably steady behavior throughout the whole simulation (Figure 2B), and the DNA tract containing the nick was completely immobilized by the protein as evidenced by the low rmsd from the average structure (Figure S2), regardless of the different loop conformations.

The P42 sequence contains two inverted and self-complementary tracts of six and eight nucleotides each that allow it to bend back onto itself and form a double-stranded stem. In this case, however, only one loop is present that comprises the six unpaired nucleotides that connect both strands of the stem. The tail of this hairpin is made up of the remaining eight nucleotides (5′-CTTCGGCG-3′) that overhang unpaired at the 3′-end (Figure 1B). The P42 hairpin’s stem showed a remarkable stability whereas loop and tail visited different conformations during the uMD simulations (Figure S3). Due to the hydrophobic effect [20], the hairpin’s tail appears to be folded as a pseudoloop that hides the bases from the solvent (Figure 2C). Thus, the equilibrated conformation of this hairpin is reminiscent of the dumbbell structure observed for D42.

### 2.2. Proposed Kissing Loop Interaction as a Pre-Annealing Step

**Figure 3:**
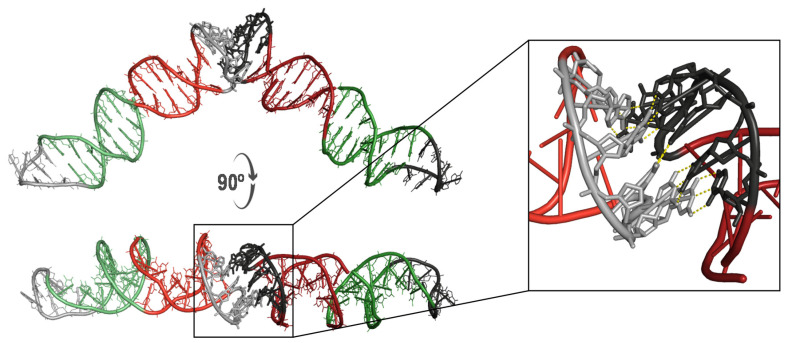
Proposed kissing loop interaction between D42 (**left**, light colors) and P42 (**right**, dark colors) ssDNA molecules. Hydrogen bonds are displayed as yellow dotted lines.

The fact that D42 and P42 sequences are fully complementary makes it possible for the two single-stranded oligonucleotides to pair by hydrogen bonding and give rise to a 42-bp dsDNA microcircle containing a single nick in the P42 strand. The most likely mechanism underlying this annealing is, in our view, through the formation of a kissing loop because six out of the eight nucleotides in the overhang of the P42 hairpin have their (unpaired) complementary bases in one of the dumbbell loops (Figure 1). We therefore postulated that the recognition of this loop by the hairpin tail could trigger a zipper-like motion by means of which the hydrogen bonds and stacking interactions of the double-stranded stems of both structures would progressively weaken. Simultaneously, the unpaired bases would then be allowed to interact with their complementary counterparts to give rise to a more stable and energetically favored dsDNA microcircle containing a single nick in the P42 strand. Experimentally, this hybridization (in ~30% yield) takes place upon incubation for 5 min at 90 °C and cooling down to room temperature over a period of 30 min. This procedure is crucial because of the need to disrupt existing intramolecular hydrogen bonds in both D42 and P42 stems and to favor formation of intermolecular hydrogen bonds. Furthermore, we show that this process involves threading of the P42 strand through the hole of a doughnut-like D42 bubble (Figure S4). The recognition of the dumbbell loop (5′-GCCGAA-3′) by the complementary bases of the hairpin’s hanging tail (3′-CGGCTT-5′) was modeled and simulated as a kissing loop because of the feasibility of canonical base-pairing hydrogen bonding interactions (Figure 3). Of note, this pre-annealing complex displayed a remarkable stability during the ensuing uMD simulation in the absence of any positional or conformational restraints.

### 2.3. Singly Nicked and Covalently Closed DNA Microcircle

**Figure 4:**
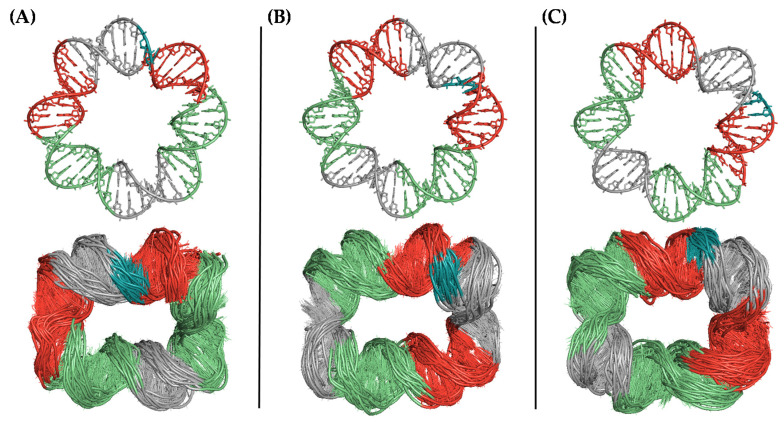
Conformational diversity of the DNA microcircle after uMD equilibration (bottom) depending on the register of the helix ((**A**) no offset; (**B**) 5-bp offset; and (**C**) 7-bp offset) in the initial model (top). The nucleotides making up the sealed gap are colored in blue.

To identify the most likely conformation of the covalently closed microcircle we generated several torsionally relaxed circular DNA molecules differing in the position of the nick with respect to the center or periphery of the ring. When the covalently closed 42-bp dsDNA microcircles were simulated, in the presence of either Mg^2+^ or Na^+^ ions, helical distortions such as kinks and bubbles were readily apparent. The location of these structural alterations was shown not to be random. Although they accumulated in distinct sequence stretches depending on the offset present in the initial structure, all the topoisomers displayed an elliptical conformation with the kinks on opposite sides of the ellipse’s longest axis (Figure 4). These results convincingly suggest that the conformation of such a dsDNA microcircle is far from being that of a canonical circle lacking any deformation. In fact, the type of localized bending that is present in our structures is reminiscent of two closely spaced U-turns, as described in the complexes of DNA with integration host factor (IHF) [21] and mitochondrial protein Abf2p [21].

These three topoisomers were also simulated with a single nick in the P42 strand to reproduce the molecule that would result from the annealing process and therefore turn into the substrate for the T4 DNA ligase. It was observed that the nicked ends remained aligned independently of the ionic environment (Na^+^ or Mg^2+^ ions) only in the case of the structure created with an initial offset of 7 bp (three replicas). Besides, the location of the kinks in the double helix coincided with those displayed by the covalently closed circles. In contrast, in the microcircles created either without an offset or with a 5-bp offset, the ends remained aligned in some replicas but not in others, although a correlation between fraying and ionic environment could not be established (Figure S5). These results suggest that the topoisomer containing an initial offset of 7 bp is the most stable and therefore the most likely candidate to be ligated into a covalently closed microcircle.

### 2.4. Post-Ligation Pre-Release DNA Microcircle

Although many different topoisomers of the nicked 42-bp microcircle are expected to appear during the annealing process, only that (or those) with a suitable conformation for recognition by the T4 DNA ligase would result in a covalently closed structure. To be a substrate for T4 DNA ligase, the singly nicked DNA microcircle must fulfil three conditions: (i) its 3D architecture has to allow the three domains of the ligase to fully embrace the stretch containing the nick, (ii) the bases and the phosphates must be suitably positioned inside the enzyme active site, and (iii) the nucleotides involved in the ligation reaction need to be properly aligned. In addition to these general prerequisites, which can be applied to any canonical dsDNA, another necessary condition can be discerned in the case of a circular dsDNA of such a small length: the nick has to be located on the outer face of the microcircle. If the gap were on the inner face, it would be inaccessible to the ligase active site due to steric constraints and the efficiency of the enzyme would be compromised. Thus, it is safe to assume that the ideal substrate for T4 DNA ligase must have the nick located on the periphery of the microcircle. Interestingly, when the three topoisomers generated differing in the position of the nick were fitted inside the enzyme as explained in the methods section, only that containing a 7-bp offset displayed an optimal architecture compatible with location of the nucleotides to be ligated inside the active site without any steric clash with the enzyme. This finding, together with the results mentioned above concerning the stability of the nicked region of this particular microcircle, makes this topoisomer the ideal candidate for ligation.

**Figure 5:**
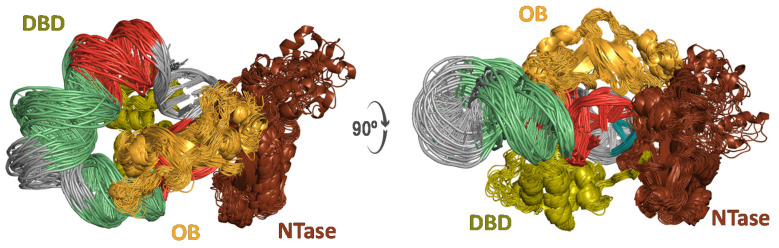
Cartoon representation from two viewpoints related by a 90° rotation about the X-axis (**left** and **right**) of T4 DNA ligase in complex with a 42-bp covalently closed microcircle. The T4 DNA ligase domains are labeled DBD (green), OBD (orange), and NTD (brown).

We therefore used the 7-bp offset microcircle, as directly provided by the MCDNA server [22] to model the complex between such a circular DNA and T4 DNA ligase, whose 3D structure was recently solved [23] bound to a canonical dsDNA molecule. It is important to note that, although a perfect circumference is not expected for such a small dsDNA microcircle, the DNA duplex region trapped inside the enzyme should not present any distortions that could interfere with the recognition and ligation processes (Figure 5). The models proposed here for the covalently closed microcircle in complex with T4 DNA ligase provide structural insight into the Michaelis complex that leads to the experimentally observed results [17].

## 3. Materials and Methods

### 3.1. Construction of the D42 Dumbbell and the P42 Hairpin

All-atom models of the D42 dumbbell and the P42 hairpin DNA molecules were built with the aid of the SimRNA web server [24], a Monte Carlo based method for predicting RNA 3D structures using sequence information. Five different clusters were generated for each molecule after introducing the corresponding RNA sequence of D42 and P42, which varied in the secondary structure assigned to each sequence stretch. The structures selected to be the initial models for D42 and P42 were the ones that reproduced the expected dumbbell and hairpin conformations, respectively, with a central base-paired stem and two single-stranded regions at both ends (Figure S6). Then, both RNA structures were transformed into DNA upon removal of the 2′-OH group. PyMOL v. 1.8 [25] was used for structure visualization, molecular editing and figure preparation.

### 3.2. Construction of the Kissing Loop

The kissing loop complex was modeled using as scaffold the crystallographic structure of an RNA kissing complex, solved at 2.9 Å resolution and deposited in the Protein Data Bank with id. 2JLT [26]. Each RNA strand was used as a template to perform best-fit superpositions of equivalent nucleotides of the single-stranded tail of the P42 hairpin and its complementary counterpart in one the single-stranded loop regions of the D42 dumbbell with the aid of PyMOL.

### 3.3. Construction of the 42-bp Microcircle

All-atom models of circular DNA molecules were built with the aid of the MCDNA web server [22], a component of the Multiscale Genomics project (https://www.multiscalegenomics.eu/MuGVRE/ (accessed on 29 April 2021)). MCDNA makes use of the NUCGEN program and the molecular manipulation language NAB [27] present in AmberTools [28], together with some in-house code developed for producing circular structures [29]. 42-bp-long DNA microcircles were built as non-writhed, torsionally relaxed structures (∆*L_k_* = 0). Given the fact that the regular pattern of position-dependent helical parameters imposed by MCDNA for building a circular DNA molecule varies depending on the definition of the 5′ end, three different starting models for each circle were created by shifting the register of the sequences by 5 or 7 bp following a previously reported protocol [29].

### 3.4. Construction of the Post-Ligation Pre-Release T4 DNA Ligase–DNA Complexes

The crystallographic structure of the enzyme in complex with an AMP-bonded dsDNA ended with a 2′,3′-dideoxyribonucleotide, solved at 2.75 Å resolution and deposited in the Protein Data Bank with id. 6DT1 [23], was used. To simulate the full-length protein, the gp45 binding loop within the NTase domain (residues 222–247), for which no density is discernible in the electron density maps, was modeled with the aid of the SWISS-MODEL web server [30]. Standard ff14SB force field [31] parameters were used for protein atoms. The AMP molecule covalently bonded to the dsDNA intermediate was replaced with a similarly bound AMP molecule, and the dsDNA intermediate was substituted with the nicked dumbbell or 42-bp covalently closed microcircle by performing best-fit superpositions of equivalent nucleotides with the aid of PyMOL. Nonbonded interactions were optimized by performing energy minimization.

### 3.5. Solvation and Electroneutrality

Each system was immersed in a truncated octahedron of equilibrated TIP3P water molecules. All the octahedra extended 12 Å away from any solute atom so that the resulting volume was enough to accommodate the D42 dumbbell (~54,000 atoms in a total volume of ~600,000 Å), the P42 hairpin (~51,500 atoms in a total volume of ~570,000 Å), the pre-annealing kissing loop (~150,000 atoms in a total volume of ~1,670,000 Å), the 42-bp microcircle (~50,000 atoms in a total volume of ~680,000 Å) and the T4 DNA ligase:DNA complexes (~68,000 atoms in a total volume of ~760,000 Å for the complex with the dumbbell and ~100,000 atoms in a total volume of ~1,225,000 Å for the complex with the DNA microcircle). Electroneutrality within the boxes was achieved by addition of enough Na^+^ or Mg^2+^ ions and ensuring a minimal separations of 4.0 Å amongst the ions and 6.0 Å between ions and DNA atoms. To avoid artifactual distortions of the double helix caused by the strong attraction of the Mg^2+^ ions by the DNA phosphate oxygens, an r^−4^ term that takes into account the ion-induced dipole interaction was added to the 12-6 Lennard-Jones model, as proposed by Li and Merz [32]. This extra parameter for Mg^2+^ was tested in a previous work by our group [29] and shown to effectively minimize the impact of this cation on the DNA architecture. The solvation and neutralization procedures described above allowed us to avoid any potential imaging issues during the ensuing molecular dynamics simulations [33].

### 3.6. Molecular Dynamics Simulations

The molecular dynamics (MD) simulations were run essentially as described [29]. Briefly, periodic boundary conditions were used and the cutoff distance for the non-bonded interactions was set at 9 Å. Electrostatic interactions were represented using the smooth particle mesh Ewald method with a grid spacing of 1 Å. The SHAKE algorithm was applied to all bonds involving hydrogens so that an integration step of 2.0 fs could be employed. The simulation protocol made use of the *pmemd.cuda_SPFP* engine implemented in AMBER 18 [28] running on a single NVIDIA GeForce GTX 1080 RTX 2080 Ti graphics processing unit (GPU). First, solvent molecules and counterions were allowed to redistribute around the positionally restrained solute (5 kcal mol^−1^Å^−2^) using energy minimization and the resulting systems were progressively heated from 100 to 300 K during 0.1 ns using the same restraints. Then, the systems were equilibrated at 300 K for 2.5 ns in the absence of any restraints and further simulated under the same conditions up to a total time of 300 ns during which system coordinates were collected every 5 ns for further analysis. Each system studied in this work was replicated three times by using different MD starting velocities that were assigned by means of a random number generator based on the current date and time. To simulate the same system under different ionic environments, the first and second replicas were neutralized with Na^+^ ions whereas the third one was neutralized with Mg^2+^ ions, thus resulting in a total simulation time of 0.9 µs.

## 4. Conclusions

The combination of molecular modeling and uMD simulations has allowed us to characterize the different DNA structures involved in the construction of the smallest, to the best of our knowledge, dsDNA microcircle reported to date [17]. Our 3D models of the single-stranded D42 dumbbell and the P42 hairpin constructs provide atomic insight into the recognition event that must take place for the DNA bases in the cohesive tail of the hairpin to pair with their complementary bases in one of the single-stranded loops of the dumbbell. We propose that a kissing loop involving six base pairs makes up the core of the nascent nicked dsDNA microcircle and suggest a feasible pathway for the hybridization of the remaining complementary bases to yield a nicked dsDNA microcircle. We also modeled the pre-ligation complex of T4 DNA ligase with the D42 ssDNA and the post-ligation pre-release complex involving the same ligase and the covalently closed D42:P42 DNA microcircle. In both cases, the 3D models displayed DNA structures compatible with enzyme recognition and ligation of the nicked ends.

The uMD simulations of different topoisomers of the covalently closed and singly nicked 42-bp microcircle provides strong evidence that the architecture of such a small DNA circle does indeed differ from the canonical 10.5 bp per helical turn, as the formation of two U-turns appears to be inescapable to release the excess of bending stress. These theoretical results are in good agreement with those obtained for longer, but also small, dsDNA minicircles [9,10,13]. We also propose the first model, to our knowledge, of a DNA microcircle in complex with the T4 DNA ligase. Our results suggest that, even though different topoisomers of the 42-bp microcircle are likely to appear during the annealing process, only those containing a particular arrangement of the nicked bases can be properly ligated. This observation can account for the relatively low efficiency of the overall microcircle generation process [17].

The description of observations on macromolecules by text and simple schemes alone is often insufficient to properly describe the underlying complexity of these systems [34]. With present-day molecular graphics and simulation tools, it is increasingly possible to convey high-quality visual information about recognition and binding events in atomic detail. Visualization of all-atom models not only helps to understand the outcome of experiments, as shown here, but also aids in the generation of new hypotheses and facilitates communication to both our peers and nonexperts.

## Data Availability

The data presented in this study are available on request from the corresponding author.

## Supplementary Materials

The following are available, Figure S1. Time evolution over the course of the uMD simulation trajectories (A) and (B) the 6 nucleotides. (C) Cartoon representation of an ensemble of 40 structures taken from the trajectory of the nicked DNA dumbbell. Figure S2. (A) Time evolution over the course of the uMD simulation trajectories (three replicas) of the root-mean-square deviations (rmsd, Å) of the central 10 base pairs of the nicked dumbbell containing the nick. (B) Cartoon representation of an ensemble of 40 structures taken from the trajectory of the nicked dumbbell in complex with T4 DNA ligase. Figure S3. Time evolution over the course of the uMD simulation trajectories (three replicas) of (A) the 30 nucleotides making up the stem and (B) the 6 nucleotides. (C) Cartoon representation of an ensemble of 40 structures taken from the trajectory of the tailed hairpin. Figure S4. Schematic representation of the annealing process by means of which the P42 DNA strand (dark color) threads through the hole of a doughnut-like D42 bubble to give rise to a singly nicked dsDNA circle with the bases making up the gap (blue) properly aligned for sealing by T4 DNA ligase. Figure S5. Conformational diversity of the nicked dsDNA microcircle after uMD equilibration depending on the register of the helix in three different replicas. The nucleotides on both sides of the gap are colored in blue. Figure S6. RNA-formatted input sequences for the SimRNA web server and the corresponding different clusters generated for (A) the D42 dumbbell and (B) the P42 hairpin (C) Cartoon representations.

## Sample Availability

Not available.
