# Supplementary material for: Structural Landscape of the Transition from an ssDNA Dumbbell Plus Its Complementary Hairpin to a dsDNA Microcircle Via a Kissing Loop Intermediate
Source: Molecules. 2021 May 19;26(10):3017. doi: 10.3390/molecules26103017 (PMC8158708; doi:10.3390/molecules26103017)
Supplement: Supplementary file 1 [file molecules-26-03017-s001.zip › molecules-1222443-supplementary.pdf]

Structural Landscape of the Transition from an ssDNA Dumbbell and its Complementary Hairpin to a dsDNA Microcircle *via* a Kissing Loop Intermediate

Alberto Mills and Federico Gago*

Área de Farmacología, Departamento de Ciencias Biomédicas, Unidad Asociada al IQM-CSIC, Universidad de Alcalá, E-28805 Alcalá de Henares, Madrid, Spain
* Correspondence: federico.gago@uah.es

Supplementary Information

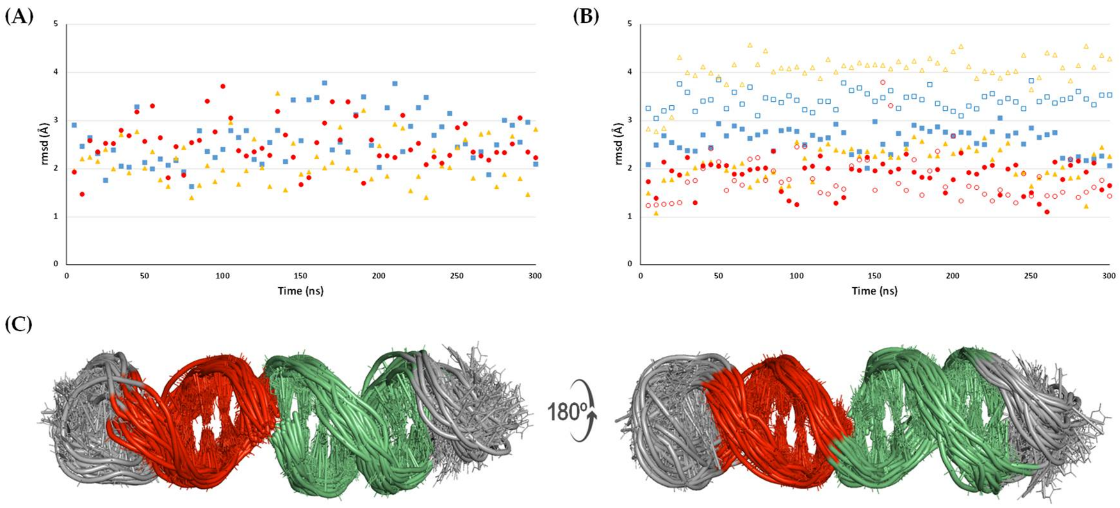

Figure S1. Time evolution over the course of the uMD simulation trajectories (three replicas) of the root-mean-square deviations (rmsd, Å) of **(A)** the 28 nucleotides making up the stem and **(B)** the 6 nucleotides of the single-stranded loop (5′-GCATAA-3′) and the 8 nucleotides (5′-CTTCGGCG-3′) of the nicked dumbbell (replica 1, blue squares; replica 2, yellow triangles, and replica 3, red dots; data points for loops 1 and 2 are shown as filled or empty symbols, respectively). **(C)** Cartoon representation of an ensemble of 40 structures taken from the trajectory of the nicked DNA dumbbell. The two views (left and right) are related by an 180º rotation about the X-axis. Each snapshot was taken every 5 ns from the post-equilibrated 100–300 ns interval of the trajectory. Self-complementary strands are colored in red and green whereas the single-stranded loop 1 (left-hand side) and loop 2 (right-hand side) are colored in grey.

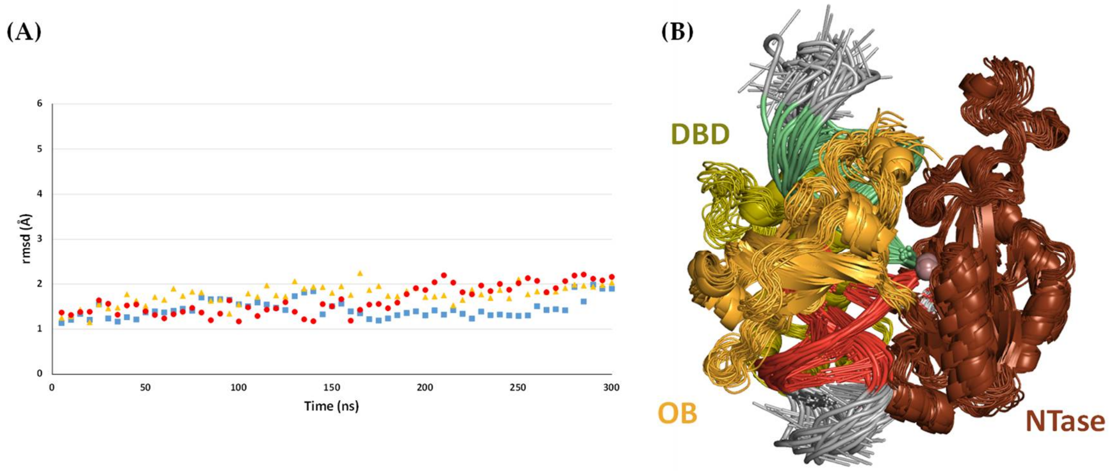

Figure S2. **(A)** Time evolution over the course of the uMD simulation trajectories (three replicas) of the root-mean-square deviations (rmsd, Å) of the central 10 base pairs of the nicked dumbbell containing the nick. **(B)** Cartoon representation of an ensemble of 40 structures taken from the trajectory of the nicked dumbbell in complex with T4 DNA ligase. Each snapshot was taken every 5 ns from the post-equilibrated 100–300 ns interval of the trajectory. Self-complementary strands are colored in red and green whereas the single-stranded loop and the 3′-tail are colored in grey. The T4 DNA ligase domains are labeled as DBD, OBD and NTase, and colored in green, orange and brown, respectively. The catalytic Mg^{2+} ion is shown as a violet sphere.

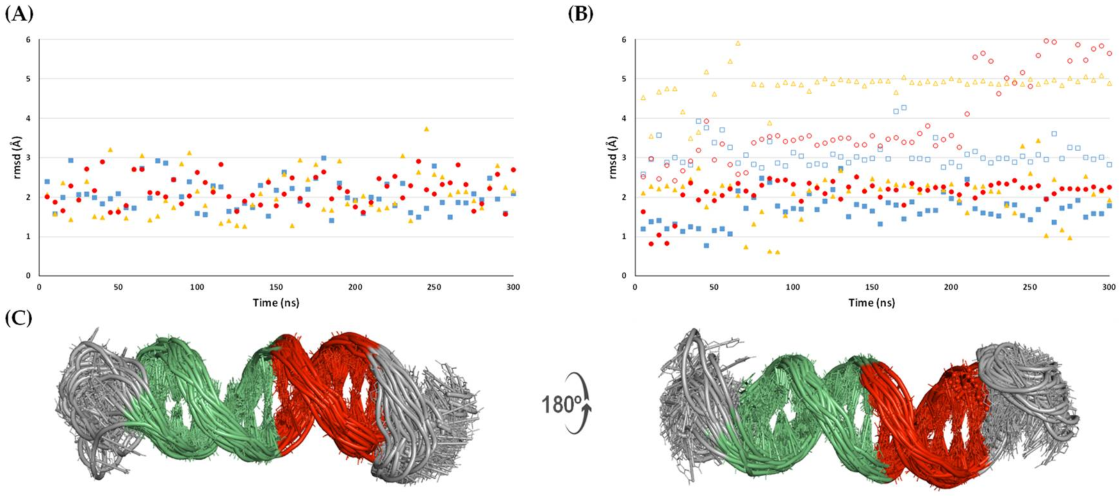

Figure S3. Time evolution over the course of the uMD simulation trajectories (three replicas) of the root-mean-square deviations (rmsd, Å) of **(A)** the 30 nucleotides making up the stem and **(B)** the 6 nucleotides of loop 1 (5′-GCCGAA-3′) and the 8 nucleotides of the single-stranded tail (5′-TTATGC-3′) of the P42 hairpin (replica 1, blue squares; replica 2, yellow triangles, and replica 3, red dots; data points for the single-stranded loop and the tail are shown as filled or empty symbols, respectively). **(C)** Cartoon representation of an ensemble of 40 structures taken from the trajectory of the tailed hairpin. The two views (left and right) are related by an 180º rotation about the X-axis. Each snapshot was taken every 5 ns from the post-equilibrated 100–300 ns interval of the trajectory. Self-complementary strands are colored in red and green whereas the single-stranded loop (left-hand side) and the 3′-tail (right-hand side) are colored in grey.

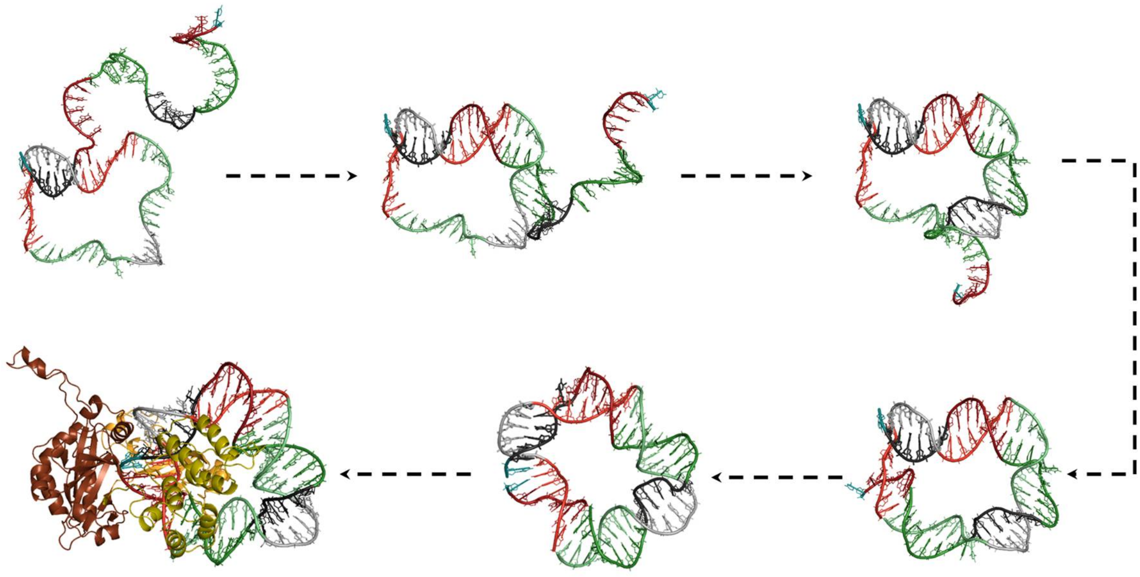

Figure S4. Schematic representation of the annealing process by means of which the P42 DNA strand (dark color) threads through the hole of a doughnut-like D42 bubble to give rise to a singly nicked dsDNA circle with the bases making up the gap (blue) properly aligned for sealing by T4 DNA ligase.

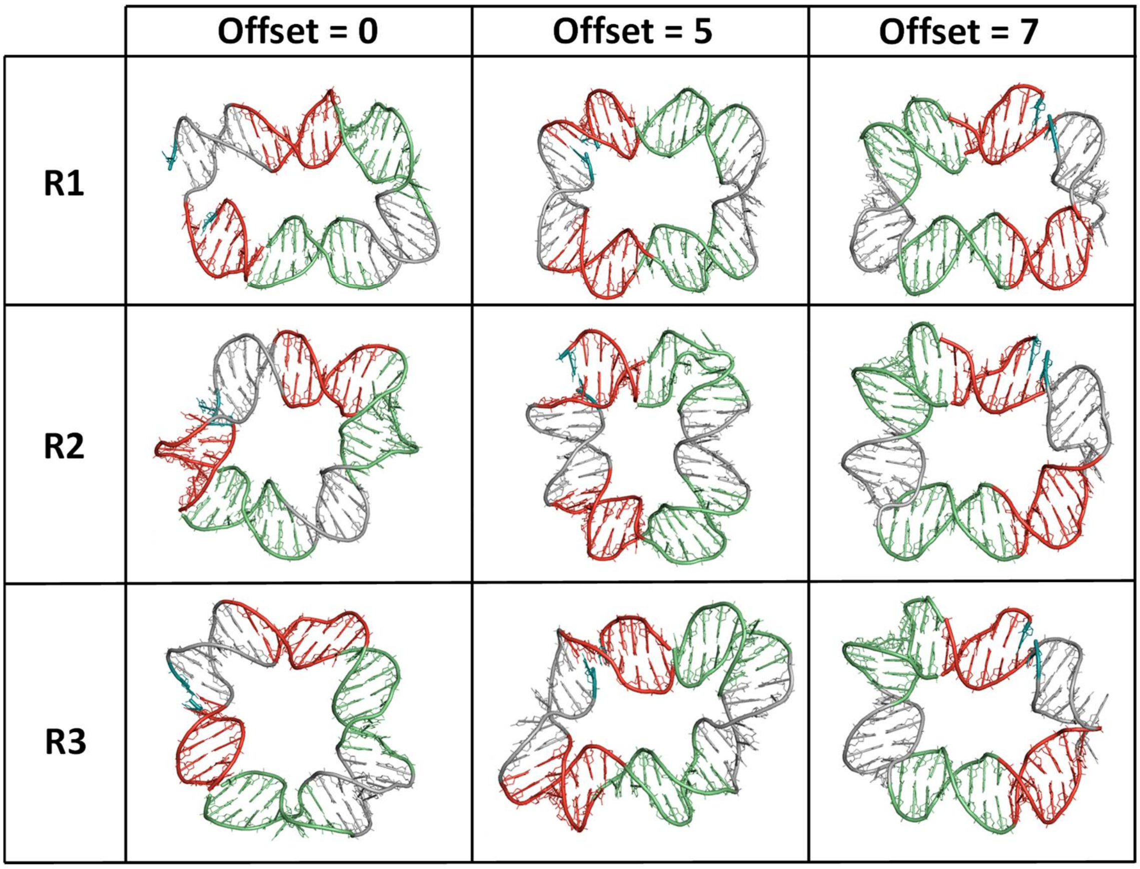

Figure S5. Conformational diversity of the nicked dsDNA microcircle after uMD equilibration depending on the register of the helix in three different replicas. The nucleotides on both sides of the gap are colored in blue.

(A)

```
5′ AUCGCGCGCCGAAGCGCGAUAUCACUCAUUAUGCUGAGUGAU 3′
> Cluster 1
(((((((......))))))).(((((((......))))))).
.......((...(..................)))........
> Cluster 2
(((((((......)))))))((((((((......))))))))
> Cluster 3
(.(((((......))))).)(((((((........)))))))
.......((.......................))........
> Cluster 4
..(((((......)))))..((((((((......))))))))
.......((.......................))........
> Cluster 5
.((((((......)))))).(((.((((......)))).)))
.......((...(..................)))........
```

(B)

```
5′ CGCGAUAUCACUCAGCAUAAUGAGUGAUAUCGCGCUUCGGCG 3′
> Cluster 1
((((((((((((((......))))))))))))))(......)
> Cluster 2
((((((((((((((......))))))))))))))........
> Cluster 3
((((((((((((((......))))))))))))))(...)...
> Cluster 4
.((((.((((((((......)))))))).)))).((...).)
> Cluster 5
.(((((((((((((......)))))))))))))((....)).
................................(........)
```

(C)

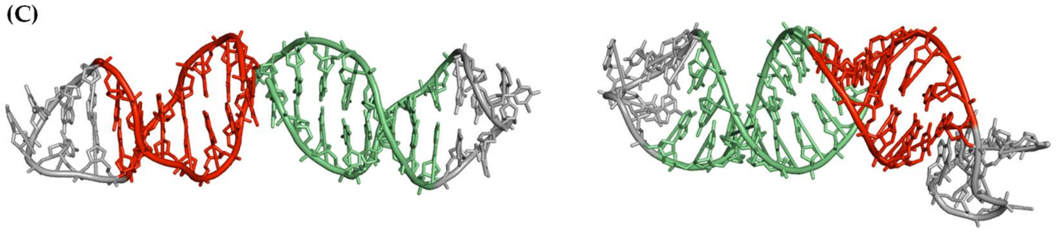

Figure S6. RNA-formatted input sequences for the SimRNA web server (https://genesilico.pl/SimRNAweb) and the corresponding different clusters generated for **(A)** the D42 dumbbell and **(B)** the P42 hairpin. A dot-bracket notation is used to represent the secondary structure of the outputs. Each character represents a nucleotide that can be base-paired (round bracket) or unpaired (dots). The selected secondary structure for each system is highlighted in bold and colored following the code used in **Figure 1** in the main text. **(C)** Cartoon representations (same color code) of the DNA-transformed initial structures of the nicked dumbbell (left) and the tailed hairpin (right).
